# Effect of Shale Powder on the Performance of Lightweight Ultra-High-Performance Concrete

**DOI:** 10.3390/ma15207225

**Published:** 2022-10-17

**Authors:** Kaizheng Guo, Qingjun Ding

**Affiliations:** School of Materials Science and Engineering, Wuhan University of Technology, Wuhan 430070, China

**Keywords:** shale powder, ultra-high-performance concrete, spread diameter, mechanical properties, microstructure, exothermic hydration

## Abstract

In this study, lightweight ultra-high-performance concrete (L-UHPC) was prepared by using SP to replace part of the cement. The main study investigated the effect of the amount of SP on the spread diameter, apparent density and mechanical properties of L-UHPC. The mechanism of the effect of SP on the hydration product of L-UHPC was studied and the pore structure of L-UHPC was also analyzed. The results show that the incorporation of SP can effectively improve the spread diameter and reduce the apparent density of L-UHPC to a certain extent. With the increase in SP content, the compressive strength of L-UHPC at 7 days of age did not change significantly. However, the compressive strengths at 3 and 28 days of age changed significantly. When the amount of SP was less than 12%, there was no significant decrease flexural and compressive strength at 28 days of age. However, the flexural and compressive strength of L-UHPC gradually decreased when the amount of SP was greater than 12%. The microstructure shows that SP can reduce the content of portlandite. This is mainly due to the fact that the addition of SP improved the stacking compactness of L-UHPC and promoted secondary hydration reactions. The content of portlandite and the hydration degree of cement were reduced. At the same time, the exothermic hydration of L-UHPC with SP was less, the hydration process was slow, and the exothermic rate of initial hydration was low. An appropriate amount of SP can effectively improve the pore structure of L-UHPC and significantly reduce the pore volume of harmful pores (50~200 nm). SP can make the L-UHPC structure more compact and has a positive effect on the development of L-UHPC strength.

## 1. Introduction

Ultra-high-performance concrete (UHPC) is regarded as the most promising engineering material in the 21st century due to its ultra high strength, high toughness, and high durability [1,2,3]. The design principle of UHPC is to enhance the particle packing density as much as possible, so a large amount of quartz sand and cement (approximately 80% of the total weight of UHPC) are used [4]. This also greatly increases the manufacturing cost [5] and limits the large-scale application of UHPC. In addition, the extremely low water–cement ratio of UHPC means that more than 50% of the cement particles are not involved in hydration and only act as a filler inside the UHPC [6,7]. Additionally, excessive unhydrated particles not only increase the cost but even cause many hazards [8]. Studies have shown that using industrial by-products [9,10,11] (silica fume (SF) [12,13], fly ash (FA) [14,15] and ground granulated blast furnace slag (GGBS) [16], etc.) instead of cement is a very effective method. The incorporation of SF, FA and GGBS can effectively reduce the amount of cement, significantly improve the strength and durability of UHPC, and improve the flowability of UHPC. However, with the wide application of UHPC, the consumption of these industrial by-products is increasing, leading to an oversupply, degradation in quality and high prices [17,18,19]. Therefore, the preparation of UHPC with composite materials [20,21] or solid waste [22,23,24] instead of partial cement has become a new research direction.

Yichang, China, has abundant shale resources and shale light aggregate production lines, which produce shale light aggregate with the advantages of light weight, high strength and low water absorption. However, the process of shale light aggregate crushing produces a large amount of shale powder (SP). In addition to using up valuable land resources, the significant SP accumulation also seriously pollutes the environment. Numerous studies on the effects of cement replacement with stone powder on the mechanical characteristics of cement-based materials have been carried out recently [25,26,27,28,29,30]. Zhang et al. [31] studies the effect of different stone powder contents (0%, 3%, 5%, 7%, 9% and 11%) on the compressive properties of concrete. The results showed that stone powder improved compressive properties of concrete. Thongsanitgarn et al. [32] investigated the effect of limestone powder (LS) particle size on the heat of hydration of Portland cement (PC) and high-calcium fly ash (FA) systems. It was found that the particle size of LS had a significant influence on the observed heat of hydration both on rate of reaction and total amount of heat release. Knop et al. [33] discovered that replacing active materials with the inert additive limestone powder could improve the performance of cement paste by increasing the surface area and the bulk density of cement particles, mainly due to the increase in bulk density when using several combinations of limestone powder with different particle size distributions. Mashaly et al. [34] found that the mortar and concrete mixes modified with granite sludge up to 20% cement replacement exhibited a negligible decline in physical and mechanical properties in addition to enhanced resistance to abrasion, freeze and thaw and sulfate attack. Xiong et al. [35] discussed the effect of different stone powders, including limestone powders, quartzite powders, and granitic powders, on the hydration behavior of silicate cement. The results of the study showed that the initial and final setting times were shortened when the limestone powders content was 5%; however, quartzite powders and granitic powders were slightly delayed. In summary, it can be seen that the existing studies mainly focus on the effect of stone powder on the mechanical properties of mortar and ordinary concrete, and there are fewer studies on the preparation of lightweight ultra-high-performance concrete (L-UHPC) based on SP.

Therefore, in this study, SP was used to replace part of the cement to prepare green low-carbon lightweight ultra-high-performance concrete. The effect of SP incorporation on the spread diameter, apparent density and mechanical strength of L-UHPC was studied. The microstructure of concrete was analyzed by XRD, TG/DTG and SEM to explain the mechanism of the action of SP in L-UHPC. Finally, the hydration process and pore structure of L-UHPC containing SP were analyzed by using the heat of hydration test and the BET test. The results of the study showed that it is feasible to use SP to replace part of cement for L-UHPC preparation. Therefore, using SP to replace part of the cement in the preparation of L-UHPC not only consumes a large amount of SP but also avoids the massive accumulation of SP to occupy land resources. At the same time, the amount of cement is reduced, and the cost of L-UHPC is reduced.

## 2. Materials and Methods

### 2.1. Materials

**Figure 1:**
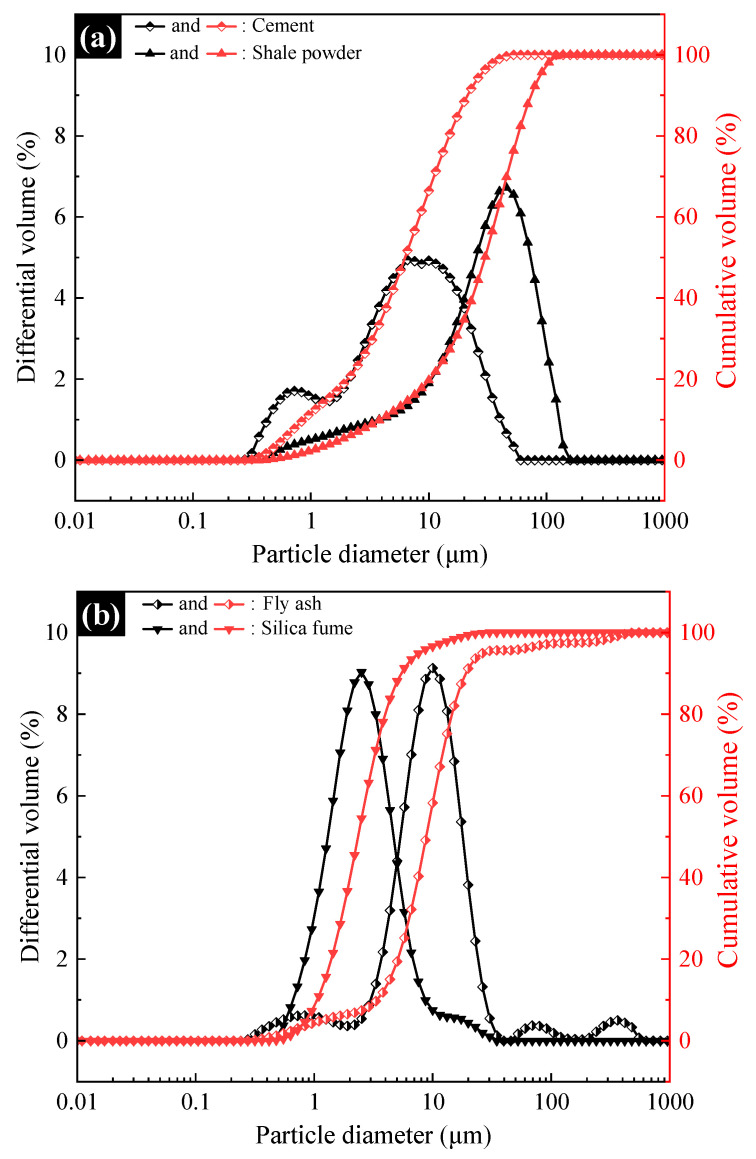
Particle size distribution curves of cement, shale powder, fly ash and silica fume. (**a**) Cement and shale powder; (**b**) fly ash and silica fume.

The cement (C) used was PII 52.5 silicate cement from China Huaxin Corporation (Bejing, China). The bulk stability of the cement is qualified, the water requirement of standard consistency is 27.53%, the loss of ignition is less than 3.5%, and the chemical composition is shown in Table 1. Figure 1a shows the particle size distribution curves of the cement, and the specific surface area and the D50 are shown in Table 2.

**Figure 2:**
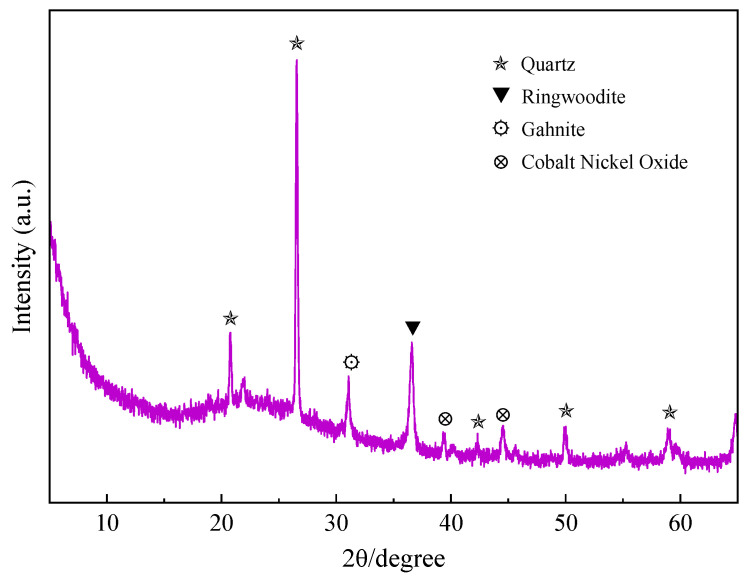
The XRD pattern of shale powder.

Shale powder (SP) was produced by a company in Tianjin, China. The specific chemical composition is shown in Table 1, the particle size distribution is shown in Figure 1a, and the specific surface area and the D50 are shown in Table 2. Figure 2 shows the XRD pattern of the SP; it can be seen that the main minerals of SP are quartz, ringwoodite, gahnite and cobalt nickel oxide.

Fly ash (FA) was produced by a company in Tianjin, China. The specific chemical composition is shown in Table 1, the particle size distribution is shown in Figure 1b, and the specific surface area and the D50 are shown in Table 2.

Silica fume (SF) was produced by a company in Chengdu, China. The specific chemical composition is shown in Table 1, the particle size distribution is shown in Figure 1b, and the specific surface area and the D50 are shown in Table 2.

**Figure 3:**
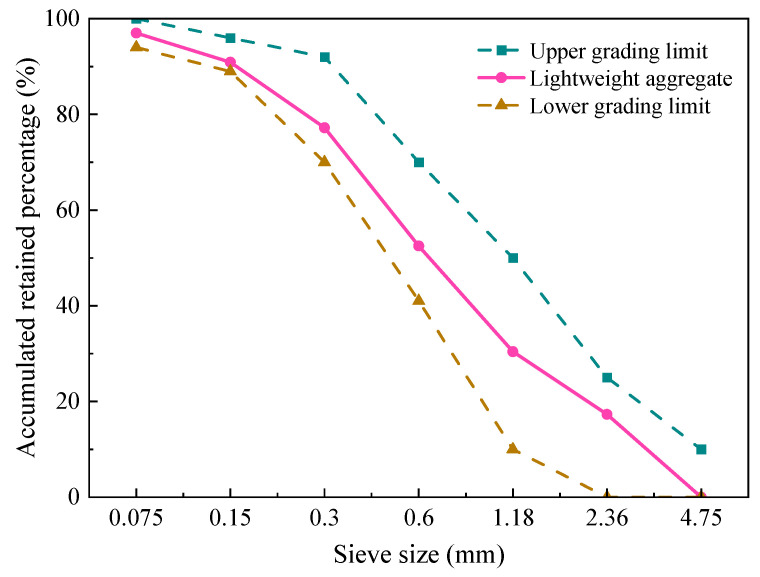
The gradation curves of lightweight aggregate.

Lightweight aggregate (LWA) was purchased from a company in Henan, China, and it was 800 grade shale ceramic sand. The particle size of LWA is 0.075~4.75 mm, the gradation curve is shown in Figure 3 and Table 3 shows the basic physical properties of LWA.

Steel fiber was purchased from a company in Tianjin, China. The length is 13 mm, diameter is 0.22 mm, density is 7.8 g/cm^3^ and tensile strength is 3000 MPa.

Water reducing agent (WRA) used is polycarboxylic acid high-performance WRA, which has a solid content of 40%, a water reduction rate of 37% and a density of 1.02 g/cm^3^.

The mixing water (W) was domestic water.

### 2.2. Sample Preparation

Six groups of lightweight ultra-high-performance concrete (L-UHPC) were designed by replacing cement (C) with shale powder (SP). The amount of SP substituted for C in the designed L-UHPC was 0%, 2%, 4%, 8%, 12% and 16%, respectively (by mass). The water–cement ratio of L-UHPC was 0.17, the water reducing agent (WRA) admixture was 3%, and the volume rate of steel fiber was 2%. The mixing proportions used for sample preparation are shown in Table 4.

**Figure 4:**
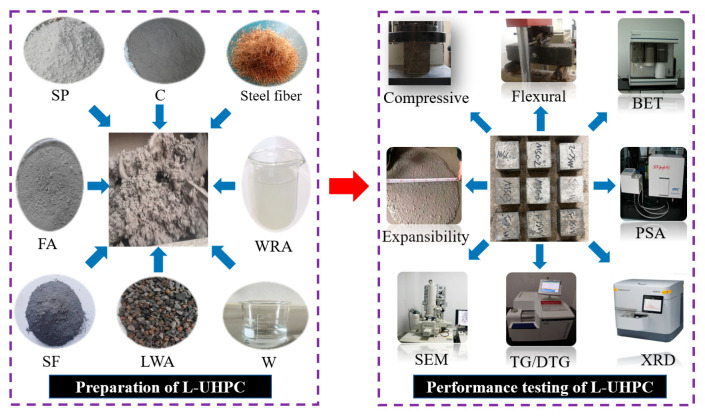
The preparation and performance testing of L-UHPC.

Before preparing the L-UHPC, the LWA was pre-wetted for 24 h, and the pre-wetted water came from the total water consumption. The specific preparation steps of freshly mixed L-UHPC are shown in Figure 4. (1) The powder materials (C, SP, FA, and SF) and the pre-wetted LWA were poured into the mixer and stirred for 1 min. (2) Added all the WRA and 80% water into the dry mixture and continued to stir for 4 min to form a slurry with certain fluidity. (3) Sprinkled the steel fiber evenly into the slurry, and added the remaining water, stirring again for 4 min, the total stirring time was 9~10 min. After mixing, the L-UHPC mixtures were poured out to test spread diameter. Later the spread diameter test was completed, the mixtures were immediately loaded into molds. Then, a vibration table was used to make mixtures dense. Finally, these samples were first covered with film for natural curing for 1d and then the molds were dismantled. Afterwards, these samples were placed into a standard curing room (curing temperature (20 ± 2) °C, relative humidity above 95%) for curing.

Based on “the Standard for Test Methods of Concrete Physical and Mechanical Properties” (GB/T 50081-2019) [30], three groups (three specimens per group) of cubic specimens (100 mm) were made for each pitch. Three groups were used to determine the compressive strength at 3 d, 7 d and 28 d. In addition, one group of prismatic specimens (100 mm × 100 mm × 400 mm) was prepared to detect the flexural strength of 28 d.

### 2.3. Testing Methods

#### 2.3.1. Performance Tests

The spread diameter test of L-UHPC was carried out according to “the national standard of the People’s Republic of China: Standard test methods for the performance of ordinary concrete mixes” (GB/T 50080-2016) [36]. Mechanical properties tests were conducted in accordance with GB/T 50081-2019 [37]. The compressive strength (loading rate of 0.5 MPa/s) of L-UHPC was tested by using a microcomputer-controlled constant loading pressure testing machine (YAW-2000, Zhongxi Yuanda Technology Co., Ltd., Beijing, China), and the flexural strength (loading rate of 0.2 kN/s) of L-UHPC was tested by using an electro-hydraulic flexural testing machine (YAS-300, Zhongxi Yuanda Technology Co., Ltd., Beijing, China).

#### 2.3.2. SEM, XRD and TG/DTG Tests

In this experiment, the samples that reached the test age were soaked in isopropanol for 24 h, and then the liquid was changed and continued to soak for 6 days to stop hydration. Then, the samples that stopped hydration were placed in a vacuum drying oven for constant weight drying. Flake samples with flat surfaces were selected, and scanning electron microscopy (SEM) tests were performed on these flake samples using JSM-IT500LV manufactured by a Hitachi Limited in Tokyo, Japan (Japanese company). The main observations were the morphology of the hydration products of cement mixed with shale powder and the interface transition zone (ITZ) between the light aggregate and the slurry. The samples were grounded into a powder with a maximum particle size of 0.08 mm. Then, the samples were analyzed by X-ray diffraction (XRD) and thermogravimetry (TG) for the composition of the physical phases and mineral phase alterations. The XRD test was performed with CuKα radiation, 40 kV tube pressure, 40 mA tube current, 0.02° step, 2°/min scan speed and 5~70° scan range. TG testing was performed in a nitrogen environment with a temperature range of 30~1000 °C and a temperature rise rate of 10 °C/min.

#### 2.3.3. BET Test and Hydration Analysis

A 2~3 mm small particle was used for the BET test. These pieces of the sample used in BET were exposed in a liquid nitrogen environment to adsorb the gas with which it contacted under different gas partial pressure. By obtaining the quality of single-layer adsorbed gas molecules on all surfaces of the material through a multipoint or single-point adsorption test, the results related to the pore structure could be calculated. This experiment mainly adopted the method of nitrogen adsorption-desorption. A TAM Air eight-channel microcalorimeter was used to test the cumulative heat of hydration and the exothermic rate of hydration in the early stage of cement paste. The heat of hydration test was carried out at 20 °C to analyze the effect of shale powder admixture on cement hydration. To reduce the test error each group of slurry was stirred with the same stirring regime (600 r/min for 30 s and 1200 r/min for 90 s), and a reference sample with the same specific heat capacity as the slurry was set to eliminate the testing error of the instrument. Each group of heat of hydration tests lasted for 5 days.

## 3. Result and Discussion

### 3.1. Effect of SP on the Performance of L-UHPC

#### 3.1.1. Spread Diameter and Apparent Density

**Figure 5:**
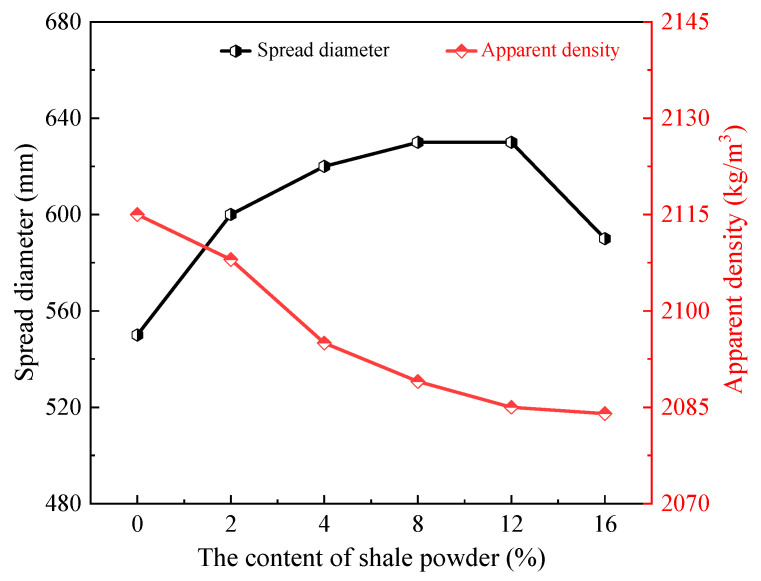
Effect of SP content on spread diameter and apparent density of L-UHPC.

Figure 5 shows the effect of SP content on the spread diameter and apparent density of L-UHPC. It can be seen that with the increase in SP content, the spread diameter of L-UHPC shows a trend of increasing and then decreasing. Without SP, the spread diameter of L-UHPC was 550 mm. When the amount of SP was increased from 0% to 12%, the expansibility of L-UHPC increased to the maximum value of 639 mm, with an increase rate of 16.2%. After that, when the amount of SP was increased to 16%, the expansibility of L-UHPC was reduced from 639 to 590 mm, which was 7% more than the expansibility of L-UHPC without SP. Overall, SP can effectively improve the working performance of L-UHPC, especially when SP is 12%, the working performance of L-UHPC is best. The main reason is that, on the one hand, as the content of SP replaces cement increases, the amount of cement decreases. At the same time, the water requirement for cement hydration decreases, and free water becomes more, so the spread diameter of L-UHPC becomes larger. However, when the content of SP is too much, the cement slurry in the L-UHPC becomes less and the lubrication is not sufficient, which makes the frictional resistance between lightweight aggregates increase, then the expansibility of L-UHPC decreases. On the other hand, SP does not undergo hydration reactions. In addition, because the particle size of SP is relatively small, a small amount of SP can play the role of dispersant in the fresh L-UHPC mix slurry, which helps the hydration of cement and thus the expansibility of the L-UHPC become larger. When excessive SP cannot play the role of dispersant, at the same time, most of the SP particles are rough and angular, they cannot play a “ball bearing” lubrication effect in the L-UHPC, so the spread diameter of the L-UHPC is reduced.

It can also be seen from Figure 5 that the apparent density of L-UHPC gradually decreases with the increase in SP content. The apparent density of L-UHPC was decreased from 2115 kg/m^3^ to 2084 kg/m^3^ when the content of SP was increased from 0% to 16%, with a decrease rate of 1.5%. The apparent density of SP is lower than the apparent density of cement, which is the cause. Thus, the apparent density of L-UHPC decreases as SP concentration increases. In conclusion, the right amount of SP can greatly enhance the spread diameter of L-UHPC as well as decrease its apparent density, but the latter effect is less pronounced.

#### 3.1.2. Mechanical Properties

**Figure 6:**
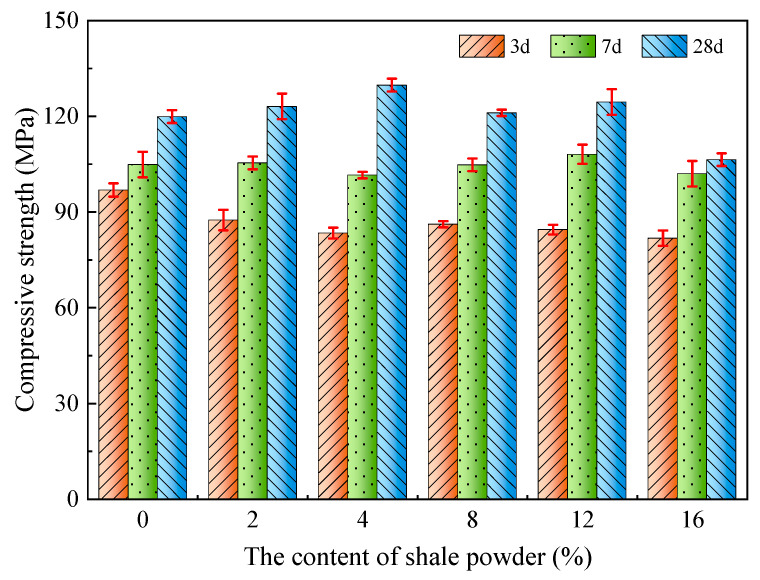
Effect of SP content on compressive strength of L-UHPC.

The effect of SP content on the compressive strength of L-UHPC is shown in Figure 6. It can be seen that with the increase in SP content, the compressive strength of L-UHPC at different ages presented inconsistent laws. At the age of 3 days, compared to L-UHPC without SP, the compressive strength of L-UHPC mixed with SP decreased. The compressive strength of L-UHPC was 96.9 MPa when the content of SP was 0%. The compressive strength of L-UHPC was 82 MPa when the content of SP was 16%, and it decreased by 15.4%. The results show that the addition of SP led to a significant decrease in the compressive strength of L-UHPC at 3d, which is not conducive to the development of early strength in L-UHPC. At the age of 7 days, when the content of SP was 0%, 2%, 4%, 8%, 12% and 16%, the compressive strengths of the L-UHPC were 104.9, 105.4, 101.6, 104.8, 108.1 and 102 MPa, respectively. The overall fluctuation range is between 2.8% and 3.1%, which indicates that the dosing of SP has little effect on the compressive strength of L-UHPC at 7d. At the age of 28 days, the compressive strength of L-UHPC tends to increase first and then decrease with the increase in SP dosing. Compared with L-UHPC without SP, when the content of SP was increased from 0% to 16%, the compressive strength of L-UHPC at 28d increased by 2.7%, 8.3%, 1.0%, 3.8% and −11.3% It shows that the compressive strength of L-UHPC at 28d increases when the amount of SP is not more than 12%. Additionally, when SP is greater than 12%, the compressive strength of L-UHPC decreases significantly. In conclusion, the moderate amount of SP has little effect on the compressive strength of L-UHPC at early ages, and it is beneficial to the development of the compressive strength of L-UHPC at 28d. However, the compressive strength of L-UHPC at all ages is significantly reduced by adding excessive SP. The optimal amount of SP in L-UHPC is 4%, and the maximum content should not exceed 12%.

The main reasons for the above phenomenon are as follows: (1) In the initial stage, after adding SP, SP is a hydrophilic material, which surrounds part of the cement particles and delays the hydration of the cement, causing the early strength of L-UHPC to decrease. As the cement hydration reaction continues, SP acts as a nucleating matrix for cement hydration products and promotes cement hydration, resulting in a gradual increase in the strength of L-UHPC. (2) The suitable content of SP can not only increase the compactness of cement stone and reduce interfacial water leakage, but also the effective accumulation of SP can densify the transition zone and strengthen the interface transition zone between aggregate and cement stone, which increases the strength of L-UHPC. The particles of SP are irregular in shape, mostly rough and angular. The particles mesh with each other, which improves the mechanical properties of L-UHPC. In addition, SP contains a small amount of CaCO_3_ for the hydration reaction of C_3_A to produce hydrated calcium carbonate [38], which prevents the conversion of AFt to AFm and ensures the development of L-UHPC strength. (3) As an inert material, SP does not undergo hydration reactions and has no cementing effect. The excess SP leads to a reduction in cement content, which causes a reduction in cement slurry and a weakening of the bond between the lightweight aggregates. Ultimately, the strength of the L-UHPC decreases.

**Figure 7:**
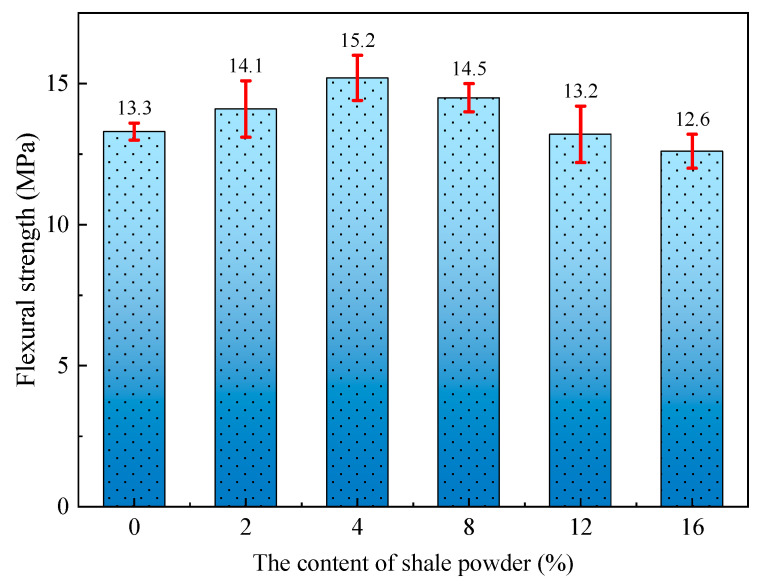
Effect of SP content on flexural strength of L-UHPC.

Figure 7 shows the effect of SP content on the 28d flexural strength of L-UHPC. From Figure 7, it can be seen that the 28d flexural strength and 28d compressive strength of L-UHPC have the same trend; both show the trend of increasing and then decreasing with the increase in SP dosing. The flexural strengths of L-UHPC were 13.3, 14.1, 15.2, 14.5, 13.2 and 12.6 MPa when the contents of SP were 0%, 2%, 4%, 8%, 12% and 16%, respectively. It is noteworthy that the flexural strength of L-UHPC reached the maximum value when SP was mixed at 4%, with an increase rate of 14.3%. When the amount of SP was 12%, the flexural strength of L-UHPC was 13.2 MPa, which was only 0.8% lower than the flexural strength of L-UHPC without SP. When the content of SP was 16%, the flexural strength of L-UHPC decreased by 5%. This indicates that an appropriate amount of SP (SP content not exceeding 12%) contributes to the development of L-UHPC flexural strength, especially the L-UHPC flexural strength is the highest when the dosing of SP is 4%. However, the excessive amount of SP is not conducive to the development of flexural strength in L-UHPC, resulting in the decline of L-UHPC flexural strength.

The changes in flexural strength of L-UHPC caused by the incorporation of SP are consistent with the reasons for the changes in compressive strength of L-UHPC. It mainly includes the filling effect, activity effect and particle morphology effect of SP [39,40]. Specifically, an appropriate amount of SP and cement together in the hydration reaction promotes the development of L-UHPC flexural strength. The particle morphology and stacking effect of SP make the frictional resistance between lightweight aggregates increase, the compact structure improves the mechanical strength of L-UHPC. However, the direct reason for the decrease in L-UHPC strength is that the excessive SP leads to the decrease in cement content, which causes an insufficient bond between the lightweight aggregates and cement stone.

### 3.2. Effect of SP on the Hydration Product of L-UHPC

**Figure 8:**
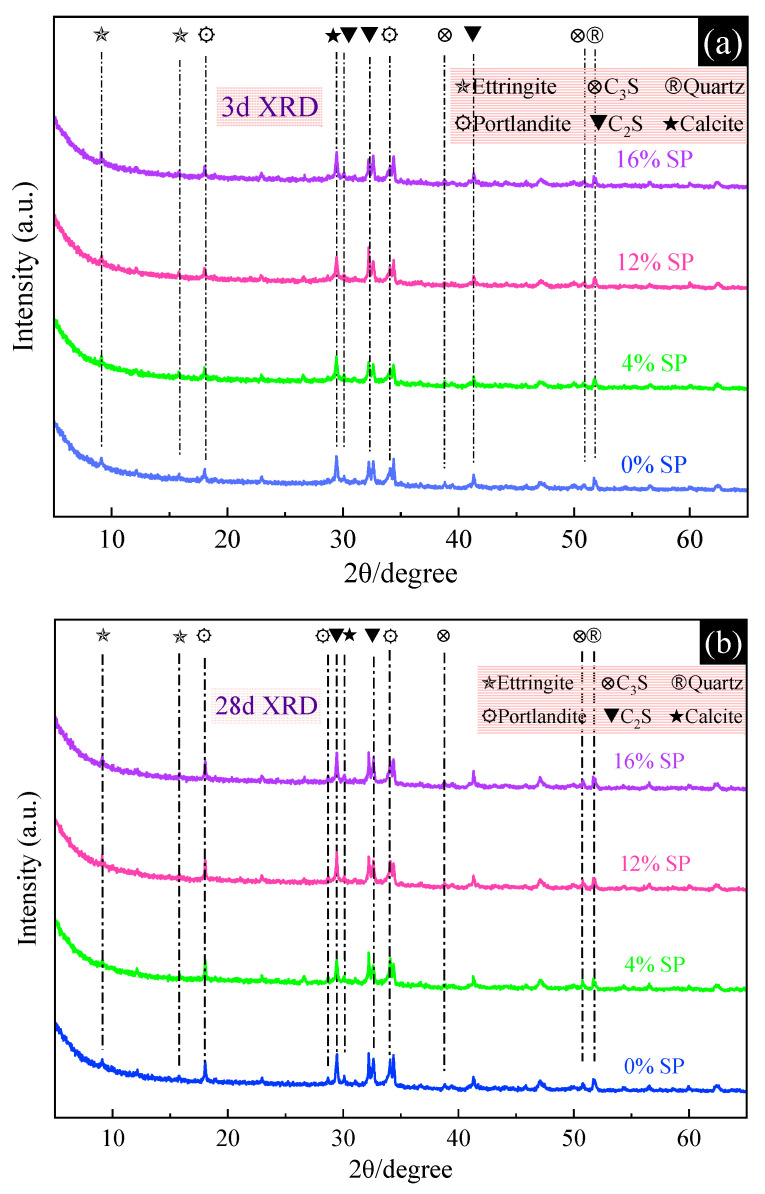
The XRD patterns of L-UHPC with different SP content. (**a**) 3 d XRD; (**b**) 28 d XRD.

Figure 8 shows the XRD patterns of L-UHPC with different SP content at 3d and 28d. From Figure 8, it can be seen that the hydration product composition of L-UHPC with different SP content is basically the same at 3 d and 28 d. Ettringite, portlandite, quartz, calcite, C_3_S and C_2_S are the most obvious crystalline phases in the XRD patterns. It shows that the incorporation of SP has no obvious effect on cement hydration products. The peak intensity of portlandite (34.2°) gradually decreased with the increase in SP amount. The gradual decrease in the peak intensity of portlandite indicates that SP has volcanic ash activity and can consume portlandite to generate more hydration products. In addition, the peak strength of ettringite in L-UHPC slightly decreased after the addition of SP. Additionally, in the early hydration process, the peak strength of ettringite (9.1°) slightly increased with the increase in SP content. It shows that the addition of SP is beneficial for the L-UHPC to be able to form more ettringite during the early hydration process. The reason is that the small amount of CaCO_3_ in SP not only reacts with C_3_A in cement to form ettringite [41], but also inhibits the conversion of ettringite formed at the early stage of hydration to AFm at the later stage. Thus, it can contribute to the improvement of the strength of cement stone at the later stage. However, with the increase in SP content, the excessive ettringite generation can destroy the microstructural integrity of cement stone, resulting in a decrease in the early compressive strength of L-UHPC. It is worth noting that there are obvious diffraction peaks of C_3_S and C_2_S in the XRD patterns of L-UHPC at 7D and 28D. This is mainly due to the fact that the cement amount in L-UHPC is high while the water cement ratio is small, and part of the cement is not hydrated. Therefore, the diffraction peaks of C_3_S and C_2_S can be clearly seen from the XRD patterns of L-UHPC.

**Figure 9:**
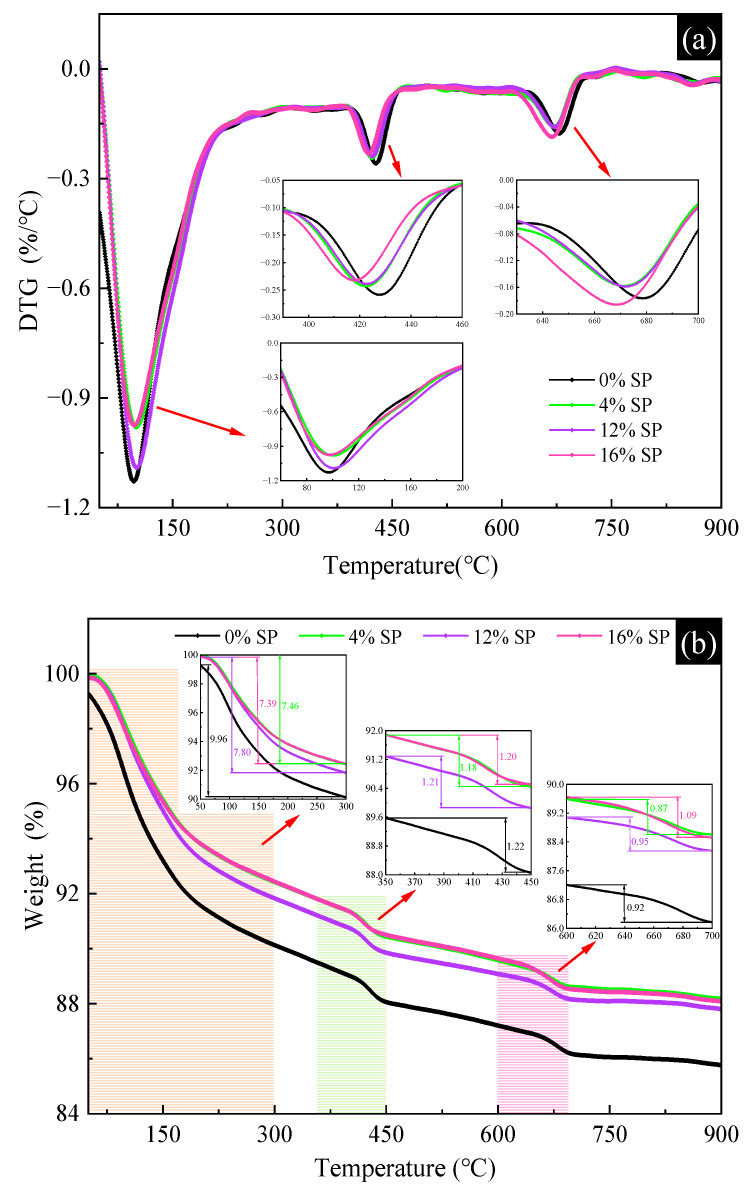
The TG/DTG curves of L-UHPC with different SP content. (**a**) DTG curves; (**b**) TG curves.

The TG/DTG curves of L-UHPC with different SP content at 28 d are shown in Figure 9. From Figure 9a, it can be seen that the decomposition of L-UHPC mainly occurs in three stages, from 0 to 300 °C, 350 to 450 and 600 to 750 °C. According to the available studies, the mass loss of the samples between 0 and 300 °C mainly includes the decomposition of ettringite, C-S-H and the water loss of other physical phases [42]. It can be seen that the addition of SP leads to a reduction in the mass loss of the specimens at this stage. This may be caused by the reduction in the amount of cement after the replacement of cement by inert SP. The mass loss of all the samples at 350~450 °C is mainly caused by the decomposition of portlandite [43]. It can be seen from Figure 9b that the mass loss of portlandite caused by calcination is less with the increase in SP content. This is consistent with the XRD analysis, which indicates that the addition of SP further reduces the production of portlandite. At the third stage (600~750 °C), the decomposition of calcium carbonate occurred mainly in the samples [44]. SP containing 16% L-UHPC had the largest mass loss of 1.09%. The calcium carbonate in the samples mainly originates from the SP. As well as during the preparation and storage of the samples, calcium hydroxide reacts with carbon dioxide in the air to produce calcium carbonate. In conclusion, L-UHPC created by partially substituting the cement with SP has a lower content of portlandite in the hydration products, as seen by both the TG/GTG results and the XRD data. It demonstrates that SP can combine with portlandite to create additional hydration products that increase the strength of L-UHPC.

**Figure 10:**
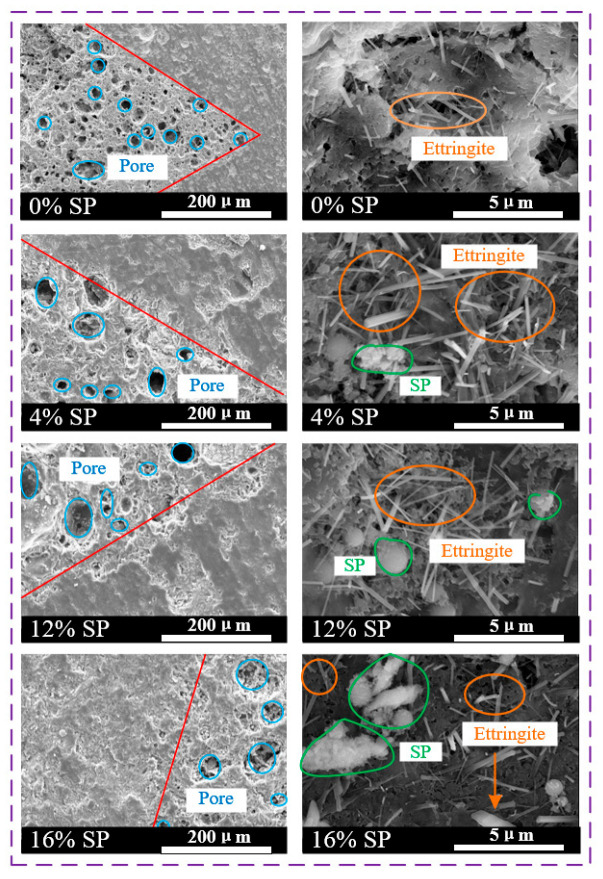
The SEM images of L-UHPC with different SP content.

Figure 10 shows the SEM images of concrete with different SP amounts at 28d. The internal structure of L-UHPC without SP was relatively loose, with more pores and cracks, and the compactness was not high. In particular, there were more micropores in the interface transition zone. It was further observed that the products after hydration of cement in L-UHPC without SP were mainly composed of different forms of C-S-H gels connected together. There are a lot of needle-rod ettringite with different lengths between the gaps of the C-S-H gel. At the same time, flakes of Ca(OH)_2_ are scattered on the surface of hydration products. With the addition of SP, it was found that the hydration products of L-UHPC mixed with SP were basically the same as those of L-UHPC without SP. They all consisted of flocculent C-S-H gel, flaky portlandite and needle-like ettringite. It is worth noting that the pores near the interfacial transition zone of L-UHPC were significantly reduced and the structure was more compact after mixing with SP. Meanwhile, with the increase in SP content, the unreacted SP in SEM images of L-UHPC increased obviously. When the SP content was 16%, the L-UHPC interfacial transition zone was the densest, but the amount of hydration products was significantly reduced. This indicates that SP can effectively fill the pores of L-UHPC, making the structure near the interfacial transition zone denser. This can promote the development of L-UHPC strength. However, the excessive amount of SP can lead to a significant reduction in hydration products in L-UHPC, which weakens the bond between the cement paste and the lightweight aggregates. This can lead to a decrease in the mechanical properties of L-UHPC.

### 3.3. Effect of SP on the Exothermic Hydration of L-UHPC

**Figure 11:**
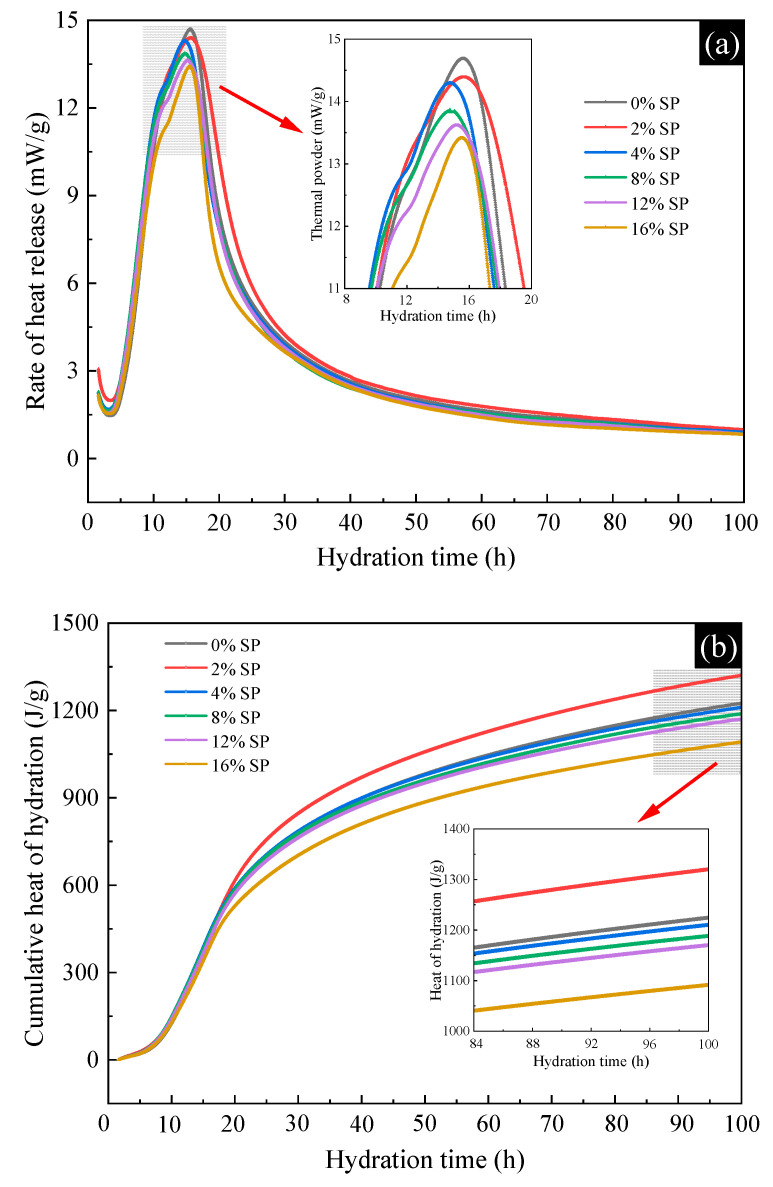
The exothermic hydration of L-UHPC with different SP content. (**a**) Rate of heat release; (**b**) cumulative heat of hydration.

The exothermic hydration curves of L-UHPC with different SP content are shown in Figure 11. It is generally believed that there are mainly two obvious exothermic peaks in the cement hydration heat curve. The first exothermic peak is caused by the rapid formation of AFt due to the first hydration of C_3_A in cement, and the second exothermic peak is caused by the rapid hydration of C_3_S starting to exotherm a lot [45]. From Figure 11a, it can be seen that all specimens have only one exothermic peak, which is mainly due to the rapid reaction of C_3_A and the short reaction time, generally within 15 min. When no SP was mixed, the peak hydration exothermic rate of L-UHPC at 12~16 h is 14.7 mW/g. As the amount of SP increases, the peak hydration exothermic rate of L-UHPC decreases gradually. The peak hydration exothermic rate of L-UHPC is 13.6 and 13.5 mW/g when the amount of SP is 12% and 16%, respectively, which is 7.5% and 8.2% lower than that of L-UHPC without SP. This indicates that the addition of SP slows down the hydration process of L-UHPC and reduces the exothermic rate of cement at initial hydration. The retardation of cement hydration is attributed to two possible reasons: one aspect is the synergistic contribution of the high water absorption of SP particles with dissolved sulfate ions. The majority of the water is first absorbed by the SP particles and lightweight aggregates, while much less water is used for the cement hydration than for the stoichiometry. On the other hand, a large amount of sulfate ions dissolved in the slurry reacts with calcium and aluminum phase to form hydration products, which adsorb on the surface of the cement particles. It slows the dissolution of cement particles and inhibits crystal growth.

From Figure 11b, it can be seen that at 100 h, the cumulative heat of hydration of L-UHPC was 1223, 1319, 1208, 11186, 1169 and 1089 J/g for the content of SP at 0%, 2%, 4%, 8%, 12% and 16%, respectively. On the whole, the addition of SP reduces the cumulative heat of the hydration of cement. When the content of SP is less than 12%, the hydration cumulative heat of L-UHPC is reduced by 54 J/g, and the reduction rate is 4%. However, when the amount of SP is more than 12%, the hydration cumulative heat of L-UHPC is reduced by 134 J/g, and the rate of reduction is 11%. It indicates that a moderate amount of SP addition has less effect on the hydration cumulative heat of L-UIHPC, while the excessive amount of SP is not conducive to cement hydration. It is noteworthy that the amount of hydration heat release of L-UHPC with 2% SP dosing was increased compared to that of L-UHPC without SP. This may be due to the fact that, on the one hand, SP has weak activity and can co-occur with cement in the hydration reaction, resulting in an increase in the hydration cumulative heat of L-UHPC. On the other hand, during the test, the slurry is not mixed uniformly or the mixing time is not enough, which causes the test error. Regarding the influence mechanism of SP on cement hydration behavior, it is generally believed that the “nucleation effect” of SP is involved [46]. SP can lower the nucleation barrier of cement particles and provide more nucleation substrates for the growth of hydration products, thereby promoting cement hydration. Following the rapid reaction of cement with water to form Ca(OH)_2_, a large amount of Ca^2+^ and OH^−^ is quickly released and diffused from the surface of the cement particles, causing the cement solution to become strongly alkaline. In cement solution, the silica-alumina oxides in SP decompose and produce new hydration products.

### 3.4. Effect of SP on the Pore Structure of L-UHPC

**Figure 12:**
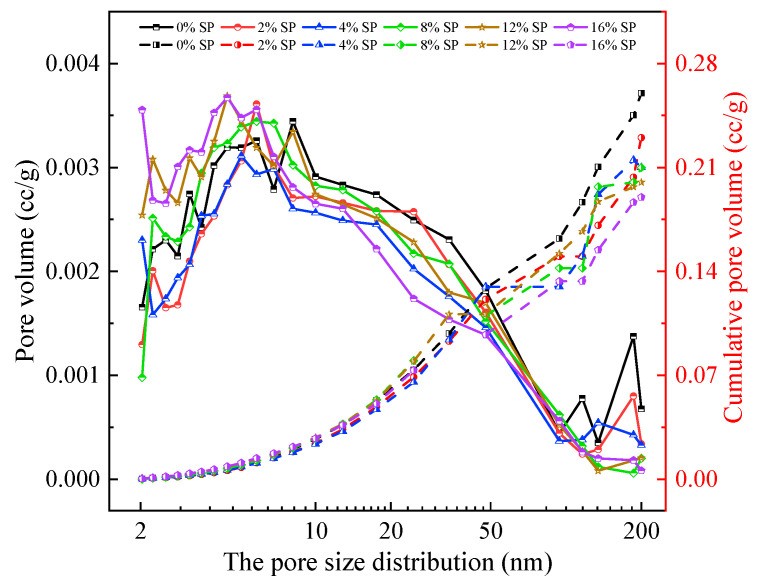
The pore size distribution and cumulative pore volume of L-UHPC.

Figure 12 shows the pore size distribution and cumulative pore volume of L-UHPC with different SP content for 28d. The peak intensities were different for all samples. For pore size less than 10 nm, the 0% SP group had two obvious spikes at 3 and 8 nm and one broad peak at 5~6 nm [47]. The 16% SP group had three obvious spikes at 3, 4 and 5~6 nm and no broad peak. Additionally, the peak intensity was larger than that of the 0% SP group. This indicates that the pore size of the 0% group is unevenly distributed at 5~6 nm and the pore size is coarsened. The pore size of the 16% SP group is more uniformly distributed but the number of small pores is relatively more. When the pore size was greater than 50 nm, the 0% SP group showed obvious spikes compared with other groups, and the peak intensity was the largest. It indicates that the L-UHPC without SP contains more pores larger than 50 nm. It can also be seen from Figure 12 that the cumulative pore volume decreases with the increase in SP content. The cumulative pore volume of the 0% SP group was 0.26 cc/g. The total pore volume of the 12% SP group was 0.2 cc/g, accounting for 77% of the 0% SP group. The cumulative pore volume of the 16% SP group was 0.19 cc/g, accounting for 73% of the 0% SP group. The results indicate that the addition of SP can optimize the pore size distribution and reduce the total pore volume of L-UHPC.

**Figure 13:**
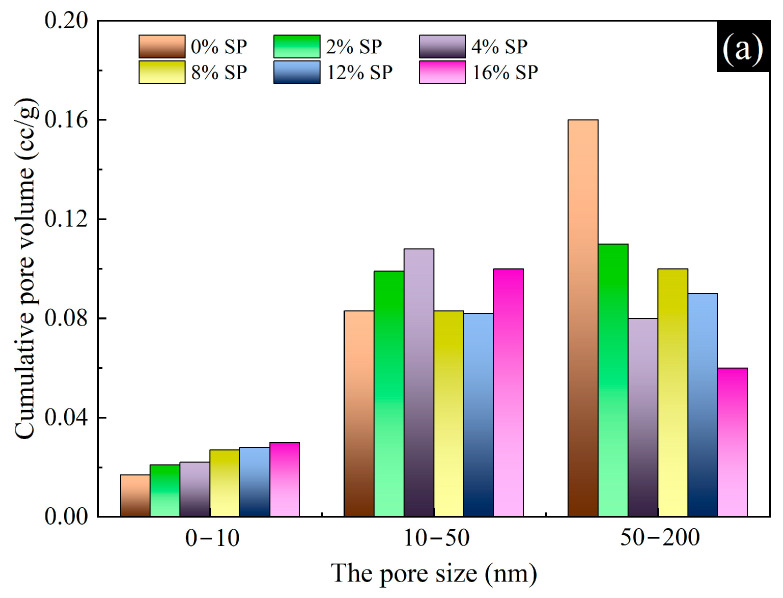

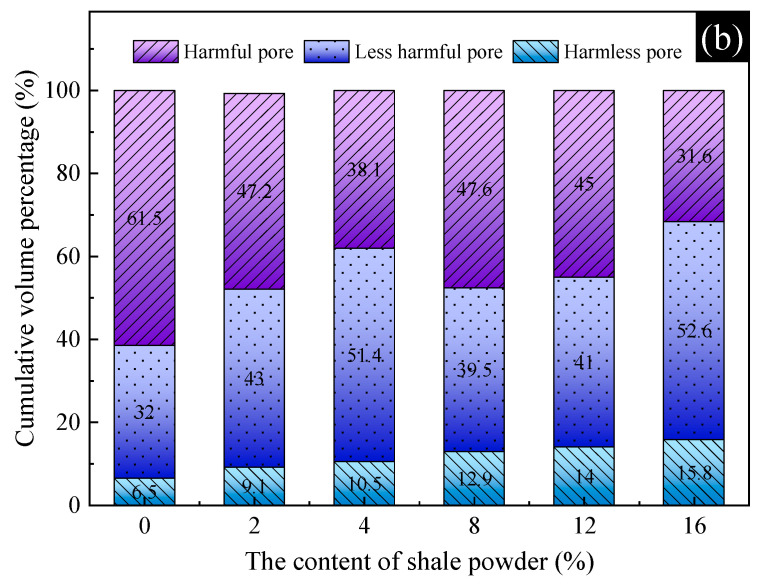
The pore structure of L-UHPC with different SP content. (**a**) Cumulative pore volume; (**b**) cumulative volume percentage.

According to the effect of pore size (pore size less than 200 nm) on the concrete properties, pores are classified into three types: harmless pores (0~10 nm), less harmful pores (10~50 nm) and harmful pores (50~200 nm). The pore volumes and pore volume percentages of L-UHPC with different SP content for 28d are shown in Figure 13. Figure 13a shows that the addition of SP made a significant difference in the pore size distribution in L-UHPC. When the pore size was 0~10 nm, the pore volume of L-UHPC increased with the increase in SP content. This is caused by the increase in SP content to reduce the amount of cement and the reduction in hydration products of L-UHPC. When the pore size was 10~50 nm, the pore volume of L-UHPC had no significant relationship with the amount of SP. With the continuous growth and diffusion of hydration products gradually filling the pores, the different degrees of hydration cause the differences in distribution of L-UHPC pores. With the increased SP amount, the harmful pore volume of L-UHPC gradually decreased. This is mainly due to the filling of fine particles in SP into the pores, which improves the pore volume and makes the L-UHPC structure denser. Figure 13b shows that the total volume of non-harmful pores in L-UHPC became larger and the total volume of harmful pores decreased when SP was incorporated. The percentage of harmful pores in L-UHPC without SP was the highest. It shows that the addition of SP can reduce the volume of harmful pores in L-UHPC, ensure the structural compactness and improve the strength of L-UHPC. The highest percentage of harmless pores was found in the 16% content of SP L-UHPC compared to other groups. This is related to the amount of cement. The high dosing of SP reduces the amount of cement and the hydration products of cement, which makes the harmless pores in L-UHPC cannot be completely filled by hydration products. In conclusion, the addition of SP contributes significantly to change the pore structure of L-UHPC. It can effectively reduce the volume of harmful pores and increase the volume of harmless pores in L-UHPC.

## 4. Discussion

In this study, the effects of SP on the workability and mechanical properties of L-UHPC were investigated. The feasibility of using SP to replace part of cement for L-UHPC preparation was discussed in terms of hydration product, exothermic hydration and pore structure. The results of this study showed that it is feasible to use SP to replace part of cement for L-UHPC preparation. The maximum substitution rate of SP was 12% and the optimum substitution rate was 4%. The effect of stone powder on the properties of ordinary concrete is consistent with the results of this study. When the stone powder substitution rate is small, the performance of concrete is slightly improved. When the stone powder substitution rate is too large, the performance of concrete decreases significantly. The use of SP to replace part of the cement in the preparation of L-UHPC not only solves the problem of SP accumulation, but also reduces the amount of cement and lowers the cost of L-UHPC. In addition, this study can provide guidance for the application of SP in L-UHPC.

However, there are limitations in the experiment. Mainly, the effect law of SP admixture on the long-term mechanical properties of L-UHPC is not clear yet. The long-term properties of SP on L-UHPC, including durability, frost resistance, drying and shrinkage, were not investigated. Therefore, subsequent studies should be conducted to investigate these aspects.

## 5. Conclusions

Based on the experimental results, the main conclusions can be presented as follows:

(a) With the increase in SP content, the spread diameter of L-UHPC first increased and then decreased, and the apparent density gradually decreased. The spread diameter and apparent density of L-UHPC were 630 mm and 2085 kg/m^3^, respectively, when the amount of SP was 12%.
(b) Compared with L-UHPC without SP, the 3d compressive strength of L-UHPC mixed with SP became smaller. The 7d compressive strength did not change significantly, and the overall fluctuation range was between 2.8% and 3.1%. Both 28d flexural strength and 28d compressive strength showed a trend of increasing and then decreasing with the increase in SP amount. When the SP content was 4%, the 28d flexural and compressive strengths of L-UHPC reached the maximum value, which was 15.2 and 129.8 MPa, respectively.
(c) With the increase in SP content, the peak strength of portlandite in the hydration products of L-UHPC gradually decreased. At the same time, the pores near the interface transition zone were significantly fewer and the pore structure was denser.
(d) The addition of SP decreased the exothermic heat of hydration, delayed the hydration process, and reduced the exothermic rate of cement in the initial hydration. However, the moderate amount of SP had less effect on the exothermic hydration of L-UHPC.
(e) The incorporation of SP optimized the pore size distribution and reduced the total pore volume in L-UHPC. The filling effect of a moderate amount of SP could effectively reduce the volume of harmful pores (50~200 nm) in L-UHPC. However, excessive dosing of SP made the cement content decrease, causing the volume of harmless pores (0~10 nm) in L-UHPC to decrease.

## Figures and Tables

**Table 1:** Chemical composition of C, SP, FA and SF (wt.%).

	SiO_2_	Al_2_O_3_	Fe_2_O_3_	CaO	MgO	SO_3_	K_2_O	Na_2_O	TiO_2_
C	19.83	4.38	3.27	64.08	1.25	2.21	0.84	0.16	0.25
SP	61.9	19.7	7.04	0.87	0	0.18	5.14	1.32	0
FA	62.6	19.71	3.72	6.28	0.97	0.13	1.36	1.22	0.67
SF	93.05	0.48	0.13	0.79	0.33	0.75	0.14	0.07	0

**Table 2:** The specific surface area and the D50 of C, SP, FA and SF.

	Specific Surface Area	D50
C	0.36	6.304
SP	0.21	28.02
FA	0.56	8.83
SF	24.52	2.35

**Table 3:** Physical performance indexes of lightweight aggregate.

	Stone Powder/%	Water Absorption/%	Cylinder Compressive Strength/MPa	Bulk Density/kg·m^−3^	Apparent Density/kg·m^−3^
LWA	2.6	10.2	6.4	740	1320

**Table 4:** Mixture proportions of the tested L-UHPC.

Sample No.	C (kg/m^3^)	SP (kg/m^3^)	FA (kg/m^3^)	SF (kg/m^3^)	LWA (kg/m^3^)	W (kg/m^3^)	Steel Fiber (%)	SP Content (%)
1	852	0	180	168	660	204	2.0	0
2	835	17	180	168	660	204	2.0	2
3	818	34	180	168	660	204	2.0	4
4	784	68	180	168	660	204	2.0	8
5	750	102	180	168	660	204	2.0	12
6	716	136	180	168	660	204	2.0	16

## Data Availability

The data that support the findings of this study are available from the corresponding author up-on reasonable request.
